# NXP032 Ameliorates Aging-Induced Oxidative Stress and Cognitive Impairment in Mice through Activation of Nrf2 Signaling

**DOI:** 10.3390/antiox11010130

**Published:** 2022-01-07

**Authors:** Jae-Min Lee, Joo Hee Lee, Min Kyung Song, Youn-Jung Kim

**Affiliations:** 1 College of Nursing Science, Kyung Hee University, Seoul 02447, Korea; sunjaesa@hanmail.net; 2 Department of Nursing, Graduate School, Kyung Hee University, Seoul 02447, Korea; lovejjoo2@naver.com; 3 Robert Wood Johnson Medical School, Institute for Neurological Therapeutics, Rutgers Biomedical and Health Sciences, Piscataway, NJ 08854, USA; ms3068@rwjms.rutgers.edu

**Keywords:** aging, ascorbic acid, cognitive impairment, DNA aptamer, oxidative stress

## Abstract

Aging is a neurodegenerative disease that leads to cognitive impairment, and an increase in oxidative stress as a major cause is an important factor. It has been reported that aging-related cognitive impairment is associated with increased oxidative damage in several brain regions during aging. As a powerful antioxidant, vitamin C plays an important role in preventing oxidative stress, but due to its unstable chemical properties, it is easily oxidized and thus the activity of antioxidants is reduced. In order to overcome this easily oxidized vulnerability, we developed NXP032 (vitamin C/DNA aptamer complex) that can enhance the antioxidant efficacy of vitamin C using an aptamer. We developed NXP032 (vitamin C/DNA Aptamin C320 complex) that can enhance the antioxidant efficacy of vitamin C using an aptamer. In the present study, we evaluated the neuroprotective effects of NXP032 on aging-induced cognitive decline, oxidative stress, and neuronal damage in 17-month-old female mice. NXP032 was orally administered at 200 mg/kg of ascorbic acid and 4 mg/kg of DNA aptamer daily for eight weeks. Before the sacrifice, a cognitive behavioral test was performed. Administration of NXP032 alleviated cognitive impairment, neuronal damage, microglia activity, and oxidative stress due to aging. We found that although aging decreases the Nrf2-ARE pathway, NXP032 administration activates the Nrf2-ARE pathway to increase the expression of SOD-1 and GSTO1/2. The results suggest that the new aptamer complex NXP032 may be a therapeutic intervention to alleviate aging-induced cognitive impairment and oxidative stress.

## 1. Introduction

Aging is known to be a major risk factor for various age-related neurodegenerative diseases including stroke, Alzheimer’s disease, and Parkinson’s disease [1,2]. Oxidative stress inevitably occurs during the aging process and plays a crucial role in progressive structural and functional damage to the brain [3]. In general, aging is associated with the progressive loss of neurons, which likely results in cognitive impairment. Age-related neuronal cell death induced by an increase in reactive oxygen species (ROS) leads to neuronal damage accompanied by excitotoxicity, microglial and astrocyte activation, inflammatory activation, and DNA damage in the brain [4]. Oxidative stress reduction in the brain may play an essential role in age-related cognitive impairment and neuronal damage [5].

In the aged brain, neurons are damaged by microglial activation and are followed by changes in neurotransmitters and the release of cytokines [6,7]. Ionizing calcium binding adapter molecule-1 (Iba-1) plays an important role in functional changes in microglia, and in many previous studies, the expression of Iba-1 is reported to be upregulated in aging-associated diseases [8]. Activated microglia are the primary source of cellular oxidative products. Glial fibrillary acidic protein (GFAP) has been used as a marker for the activation of astrocytes in brain injury, and an increase in the expression of GFAP with aging indicates that astrocytes have become reactive forms [9].

Lamin A is a major component of the nuclear membrane, mainly located below the inner surface of the nuclear membrane. The properties of lamin A regulate gene expression by constituting chromatin in the nucleus [10,11]. Lamin A increases the mRNA levels of inflammatory cytokines when overexpressed as part of the aging phenotype [12]. It has been reported that the abnormal morphology and activity of lamin A accelerates the accumulation of DNA damage and cellular senescence and increases ROS production [13]. Mechanisms by which ROS production induces cell damages include the peroxidation of polyunsaturated fatty acids in cell membranes, DNA mutations, nitrification, and carbonylation of proteins and lipids [14]. The main products of peroxidation are the 3-carbon dialdehyde species, malondialdehyde, and 4-hydroxy-nonenal (4HNE) [15]. 4HNE is used as a biomarker for oxidative stress in the brain, and it increases with cognitive dysfunction in neurodegenerative diseases. Several pathophysiological conditions associated with persistent oxidative stress, such as aging, have been reported to downregulate H2AX proteins [16]. Phosphorylation of histone H2AX (γH2AX) is an early response to DNA damage and plays a central role in DNA repair and chromosomal stability [16,17].

Vitamin C, also known as ascorbic acid, is used to prevent and treat tissue damage by reducing oxidative damage, is an essential nutrient present in a variety of foods, and is widely used as a dietary supplement [18]. Ascorbic acid, a ubiquitous water-soluble antioxidant and a cofactor for several enzymes, inhibits oxidative stress and effectively protects nerves by removing reactive oxygen species and nitrogen-based radical species generated during cellular metabolism [19]. Although ascorbic acid plays an important role in inhibiting ROS in the brain and preventing oxidative stress-related diseases, it is easily oxidized and loses its antioxidant activity. Emerging as an important source of new therapeutic molecules to enhance antioxidant activity in the brain, aptamers are highly stable substances produced by multi-purpose chemical synthesis in relatively small amounts. We developed Aptamin C320, a DNA aptamer that explicitly binds to ascorbic acid and inhibits the oxidation of ascorbic acid for a long time, and we confirmed the inhibition of oxidative stress in several situations in our previous studies [20,21].

The nuclear factor erythroid 2-related factor 2 (Nrf2)-antioxidant response element (ARE) pathway is known to induce the expression of several cytoprotective factors such as antioxidant and anti-inflammatory enzymes [22]. Nrf2 is a transcription factor that is considered a master regulator of cellular redox homeostasis. Nrf2 is inactivated by binding to Kelch-like ECH-related protein 1 (Keap1), a skeletal actin-binding protein. Nrf2 protects neurons by transcriptionally upregulating antioxidant enzyme genes that suppress oxidative stress. Several previous studies have demonstrated that oxidative stress, neuroinflammation, and neuronal injury can be prevented by increasing the activation of the Nrf2-related pathway [23,24]. The purpose of this study was to evaluate the effects of NXP032 on oxidative stress and cognitive dysfunction induced by aging by activating the Nrf2-ARE pathway.

## 2. Materials and Methods

Six-week-old female C57BL6 mice (19 ± 3 g, Orient Bio, Seongnam, Gyeonggi-do, Korea) and 17-month-old female C57B216J mice (40 ± 10 g, Korea Basic, The Science Institute, Buk-gu, Gwangju, Korea) were used for the present study. Animals were housed in a regulated environment (22 ± 2 °C, humidity of 50%) with a controlled day and night cycle (12 h of light and 12 h of dark), and food was freely consumed. This study followed the guidelines for the management and use of laboratory animals of the National Institutes of Health, and was approved by the Institutional Animal Care and Use Committee of Kyung Hee University (KHSASP-20-113).

### 2.1. Vitamin C + Aptamin C320 Preparation

Aptamers consist of single-stranded DNA or RNA-based oligonucleotides capable of selectively binding to the target molecule, and are isolated by the SELEX method [21]. The purified DNA aptamer (Aptamin C320), obtained from Nexmos Co., Ltd., is dissolved in distilled water at 95 °C for 5 min and then cooled slowly at room temperature to form a tertiary structure. Aptamin C320 was mixed with ascorbic acid (Sigma-Aldrich, MO, USA) in a ratio of 50:1 (*w*/*w*) to produce final NXP032 (200 mg/4 mg/kg of ascorbic acid: Aptamin C320).

### 2.2. Experimental Design

The 6-week-old female C57BL6 mice and 17-month-old female C57B216J mice were randomly divided into five groups (*n* = 40): young (young), aging (A + Vehicle), aging + vitamin C (A + Vit C), aging + Aptamin C320 (A + Apt C320), and aging + vitamin C: Aptamin C320 (A + NXP032). Vehicle, vitamin C, Aptamin C320, and NXP032 were administered simultaneously daily by gavage for 8 weeks. After treatment, a cognitive behavioral experiment was conducted for 2 weeks, and the mice were sacrificed the day after all behavioral tests were completed.

### 2.3. Behavior Tests

#### 2.3.1. Novel Object Recognition Test

A novel object recognition test was used to measure recognitive memory in mice, based on their behavior in exploring novel object than familiar objects. This test is performed in a familiar square box (60 × 60 × 40 cm) with high walls without spatial cues. The day before this test, mice were allowed to freely explore space in an empty square box for 10 min. The first day of the test was a training session where two identical objects (object A) were placed in the upper right and left quadrants box and the mouse was allowed to freely explore the object for 20 min. On the second day, as a test session, one of the two objects was replaced with a novel object (object B) and the mouse was allowed to freely explore the object for 10 min. Using the video recorded for each session, we analyzed the time the mice explored each object. When the distance between the mouse’s nose and the object was ≤1, this was interpreted as the mouse showing interest. The following formula below was used to calculate the discriminant index (DI).

DI = A/A + B

A: time exploring the familiar object

B: time exploring the novel object

#### 2.3.2. Radial 8-Arm Maze Test

The radial 8-arm maze test was performed to evaluate spatial learning and memory. The radial 8-arm maze apparatus consisted of a central octagonal platform (30 cm in diameter) and eight radiated and equally spaced arms (50 cm long and 10 cm wide). A small piece of food was placed at the end of the arm. All mice participating in the experiment were fasted for at least 8 h before the start of the test. On the training day, the mice were placed in the central octagon and they visited all eight arms for food. An entry into each arm was recorded and re-entry into a previously visited arm was recorded as an error. The test was terminated when mice entered all arms or when 8 min had elapsed. 

### 2.4. Immunohistochemistry and Immunofluorescence

Brain sections were carefully processed using the free-floating method, and target protein was visualized using immunohistochemistry. Firstly, the sections were washed with 0.05 M PBS and incubated with blocking solution consisting of 1% bovine serum albumin (BSA) and 10% normal rabbit or goat serum in 0.05 M PBS for 2 h at RT. Neuronal nuclei (NeuN; 1:1000; Abcam, Cambridge, UK), Iba-1 antibody (1:500; Abcam, Cambridge, UK), anti-glial fibrillary acidic protein (GFAP; 1:1000; Abcam, Cambridge, UK), lamin A (1:500; Abcam, Cambridge, UK), 4 hydroxynonenal (4HNE; 1:200; Alpha Diagnostic International, San Antonio, TX, USA), and phospho-histone H2AX (γH2AX; 1:500; Cell Signaling Technology, Danvers, MA, USA) were used as primary antibodies, and the sections were incubated overnight at 4 °C. After washing with 0.05 M PBS, it was incubated with anti-mouse, goat, and rabbit secondary antibodies (1:200; Vector Laboratories, Burlingame, CA, USA) for 2 h at RT. The bound secondary antibody was amplified using a Vector Elite ABC kit^®^ (Vector Laboratories, Burlingame, CA, USA) for 1 h at RT. For visualization, 3.3′-Diaminoben-zidine tetrahydrochloride (DAB kit; Vector Laboratories, Burlingame, CA, USA) was applied for several minutes, rinsed with deionized water. Stained sections were mounted on gelatin-coated slides and air-dried overnight at RT. Coverslips were mounted on slides using Permount^®^ (Vector Laboratories, Burlingame, CA, USA). For immunofluorescence, sections were washed with 0.05 M PBS and incubated with blocking solution consisting of 2% bovine serum albumin and 10% normal rabbit and goat serum in 0.05 M PBS for 2 h at RT. Sections were incubated overnight at 4 °C with NeuN (1:1000; Abcam, Cambridge, UK), γH2AX (1:500; Cell Signaling Technology, Danvers, MA, USA), Nrf2 (1:1000; Abcam, Cambridge, UK), and Keap1 (1:500; Abcam, Cambridge, UK). After washing with 0.05 M PBS, sections were incubated with a mixture of Alexa Fluor 488-conjugated donkey anti-rabbit IgG (1:1000; Molecular Probes, Eugene, OR, USA) and Alexa Fluor 594-conjugated goat anti-mouse or goat IgG (1:1000; Molecular Probes, Eugene, OR) for 1 h at RT. Slides were photographed using a confocal microscope (Zeiss LSM 700; Zeiss; Oberkochen, Germany). For quantitative analysis of immunohistochemistry and immunofluorescence data, the data were analyzed using Image-Pro^®^ Plus software (Media Cybernetics, Silver Spring, MD, USA) and ImageJ software (National Institutes of Health, Bethesda, MD, USA).

### 2.5. Western Blot

Proteins isolated from the hippocampus were prepared using lysis buffer (RIPA, Thermo Fisher Scientific, Waltham, MA, USA). Protein concentration was measured using a colorimetric protein assay kit (Bio-Rad, Hercules, CA, USA). Proteins (20 µg) were separated on a 10% SDS-polyacrylamide gel and transferred to polyvinylidene fluoride (PVDF). The membranes were blocked with 5% skim milk in Tris-buffered saline and 0.1% Tween 20 (TBST; 10 mM Tris, pH 7.6, 150 mM NaCl, 0.1% Tween 20)) for 1 h at RT. After washing with TBST, they were incubated with SOD-1 (1:1000; Santa Cruz, CA, USA), GSTO1/2 (1:1000; Santa Cruz, CA, USA), and β-actin (1:100,000, Santa Cruz, CA, USA) overnight at 4 °C. The membranes were incubated with horse radish peroxidase-conjugated anti-rabbit, mouse, and goat secondary antibodies (1:2,000; Molecular Probes, OR, USA) for 1 h at RT. Band detection was performed with imaging systems (ChemiDoc, Bio-Rad) using chemiluminescence Western blotting detection system (Clarity™ Western ECL Substrate, Bio-Rad, Hercules, CA, USA) for visualization. To compare relative protein expression, we analyzed using Image-Pro^®^ Plus software (Media Cybernetics, Silver Spring, MD, USA) and ImageJ software (National Institutes of Health, Bethesda, MD, USA).

### 2.6. Statistical Analysis

Experimental data are expressed as mean ± standard error of the mean (S.E.M.). SPSS version 26.0 (IBM Corp., Chicago, IL, USA) was used for statistical analysis. Statistical analysis was performed using one-way ANOVA followed by Scheffe’s post hoc test, and significance was determined as *p* < 0.05.

### 2.7. Tissue Preparation

Mice were sacrificed under deep anesthesia and perfused with ice-cold 50 mM phosphate-buffered saline (PBS) to remove blood. Brains were collected after fixation with 4% paraformaldehyde (PFA) in 100 mM phosphate buffer (PB). Whole brains were post-fixed in 4% PFA and then soaked in a 30% sucrose solution for 3 days to dehydrate. Afterward, the brains were cryosectioned 30 µm thick coronal sections using a microtome (CM3050S, Leica, Nussloch, Germany) in a cryostat (−20 °C).

## 3. Results

### 3.1. NXP032 Alleviates Aging-Induced Cognitive Impairment

**Figure 1:**
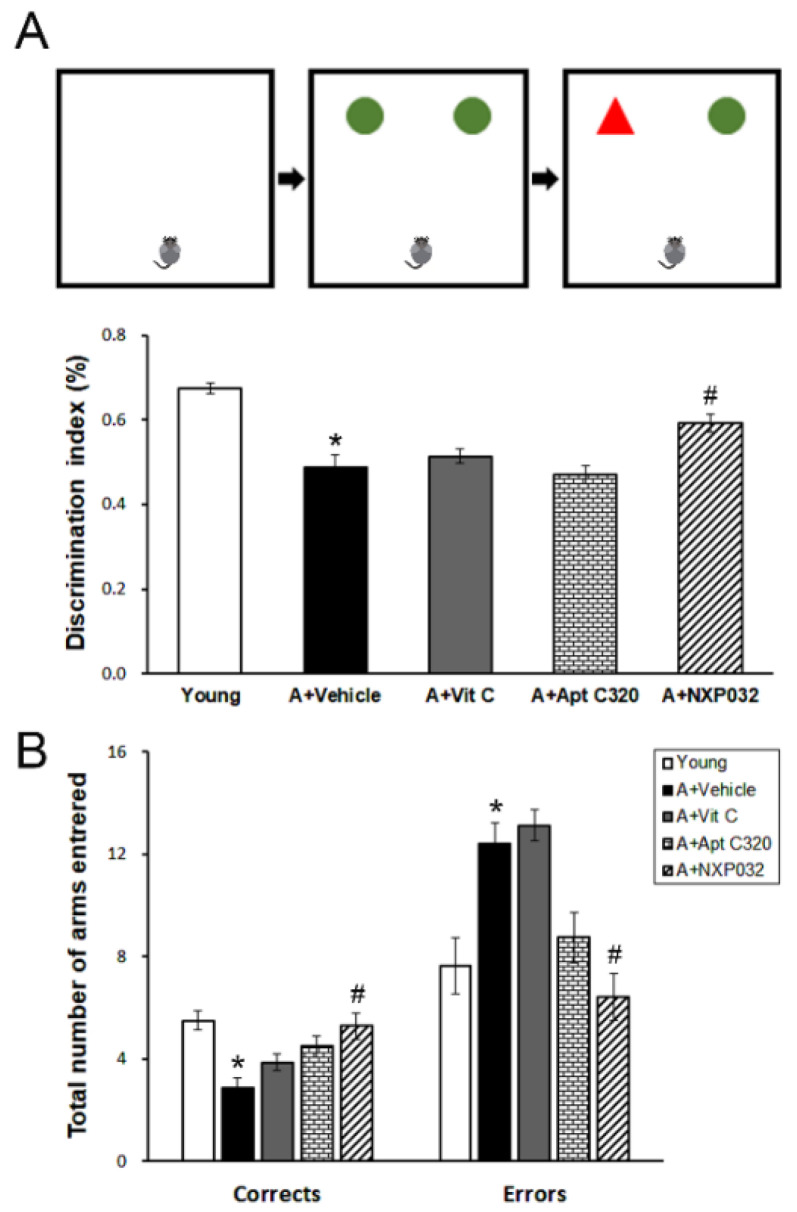
Effect of NXP032 on aging-induced cognitive impairment using the novel object recognition and radial 8-arm maze tests. (**A**) In the novel object recognition test, aged mice had significantly reduced discrimination index (%) compared to young mice. NXP032-treated mice show significantly improved discrimination index (%) compared to the vehicle-treated mice. (**B**) In the radial 8-arm maze test, aged mice had a significantly reduced number of correct choices and an increased number of error choices compared to young mice. NXP032-treated mice show a significantly improved number of correct choices compared to the vehicle-treated mice. NXP032-treated mice had a significantly lower number of error choices. The data are presented as the mean ± S.E.M. * *p* < 0.05, compared to the young group. # *p* < 0.05, compared to the aging (vehicle) group.

The novel object recognition and radial 8-arm maze tests were used to confirm aging-induced cognitive decline and to evaluate the effect of NXP032 administration. The aging (vehicle) group showed a decrease in cognitive function in memory behavioral tests compared to the young group, and the NXP032-treated group showed improved cognitive function compared to the vehicle-treated group. In the new object recognition test (Figure 1A), the NXP032-treated group showed a significantly higher discrimination index (%) than the vehicle-treated group (F = 18.074, *p <* 0.001). In the results of the radial 8-arm maze test (Figure 1B), the NXP032 group made more correct choices than the vehicle-treated group, demonstrating better spatial learning and memory functions (F = 6.519, *p <* 0.001). The NXP032 group had a significantly lower number of error choices than the vehicle-treated group (F = 10.634, *p <* 0.001).

### 3.2. NXP032 Inhibits Aging-Induced Neuronal Cell Death in the Brain

**Figure 2:**
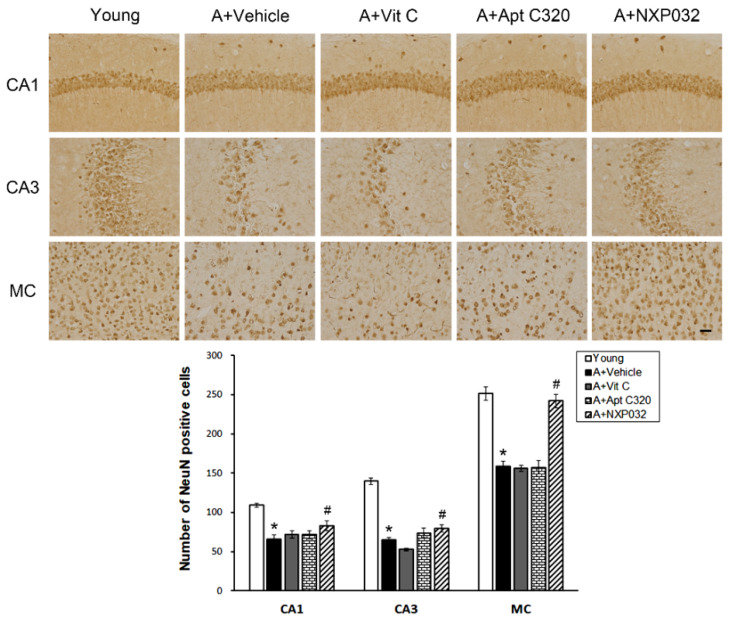
Effect of NXP032 on aging-induced neuronal cell death in the brain. Representative photographs of immunostaining of NeuN-positive cells in the hippocampal CA1, CA3, and MC regions. The aging mice had a significantly reduced number of NeuN-positive cells in the hippocampal CA1, CA3, and MC regions compared to the young mice. NXP032-treated mice had a significantly higher number of NeuN-positive cells in the hippocampal CA1, CA3, and MC regions than vehicle-treated mice. In the vit C and Aptamin 320-treated groups, the number of NeuN-positive cells did not increase in hippocampal CA1, CA3, and MC regions. The data are presented as the mean ± S.E.M. * *p* < 0.05, compared to the young group. # *p* < 0.05, compared to the aging-vehicle group. Scale bar: 50 μm.

We confirmed the expression of NeuN, which is used as a neuronal marker to confirm the neuronal cell death in the hippocampal CA1, CA3, and motor cortex regions induced by aging. The hippocampal CA1, CA3, and motor cortex regions participate in spatial attention and working memory and are important for memory exploration and formation. NeuN immunostaining in the hippocampal CA1, CA3, and motor cortex (MC) regions is shown in representative micrographs in Figure 2. The aging (vehicle-treated) group had significantly reduced NeuN-positive cells in the hippocampal CA1, CA3, and MC regions compared to the young group. The NXP032-treated group had increased number of NeuN-positive cells in the hippocampal CA1 (F = 11.644, *p <* 0.001), CA3 (F = 70.300, *p <* 0.001), and MC (F = 42.379, *p <* 0.001) regions compared to the vehicle-treated group.

### 3.3. NXP032 Inhibits Aging-Induced Activation of Microglia and Astrocytes in the Hippocampus

**Figure 3:**
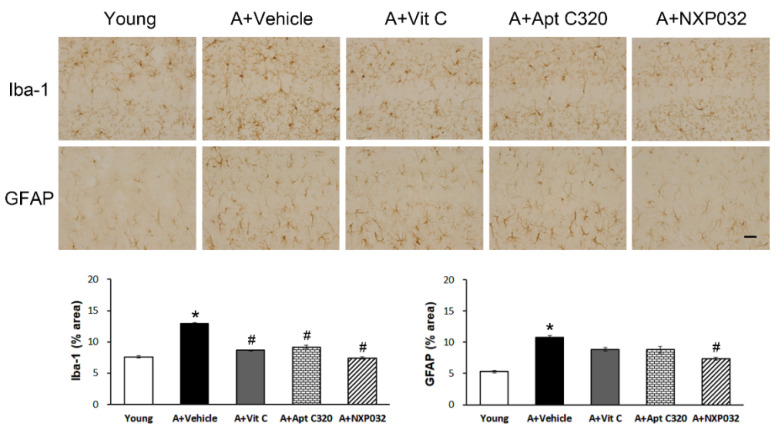
Effect of NXP032 on aging-induced microglial activation in the hippocampus. (**Upper**) Representative photographs of immunostaining of Iba-1 in the hippocampal CA1 region. (**Lower**) Representative photographs of immunostaining of GFAP in the hippocampal CA1 region. The data are presented as the mean ± S.E.M. * *p* < 0.05, compared to the young group. # *p* < 0.05, compared to the aging-vehicle group. Scale bar: 50 μm.

Activation of microglia and astrocytes has been reported to be increased in cases of injury and stress in the central nervous system. We evaluated the effect of NXP032 on aging-induced activation of microglia (Iba-1) and astrocytes (GFAP) in the hippocampus using immunohistochemistry (Figure 3). Aging-induced mice were also shown to have increased expression of Iba-1 and GFAP in the hippocampus compared to young mice. We measured the percentage area of Iba-1 expression in the hippocampal CA1 region. The aging group showed significantly increased Iba-1 and GFAP expression in the hippocampus. Iba-1 activation was decreased in all vit C, Aptamin C320, and NXP032 groups (F = 140.202, *p <* 0.001). In contrast, GFAP expression was decreased only in the NXP032 group (F = 11.543, *p <* 0.001).

### 3.4. NXP032 Inhibits Aging-Induced Lamin A Expression in the Brain

**Figure 4:**
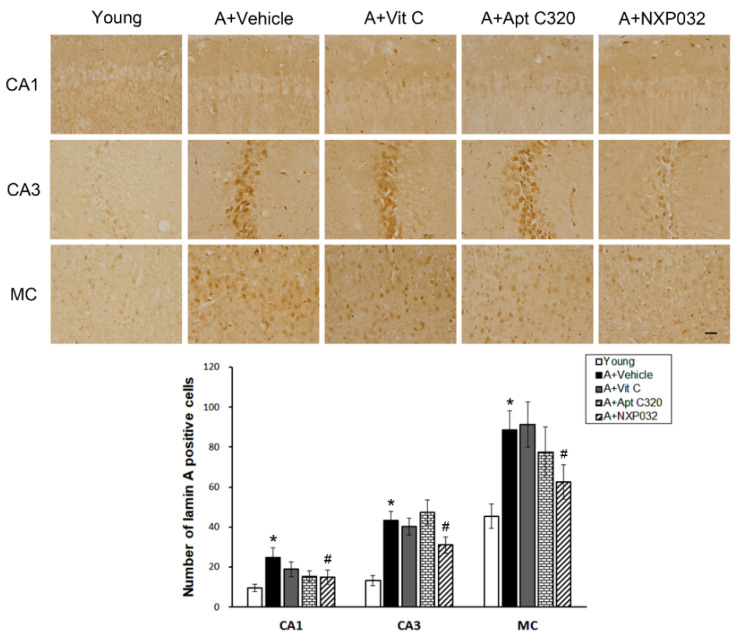
Effect of NXP032 on aging-induced lamin A in the brain. Representative photographs of immunostaining of lamin A in the hippocampal CA1, CA3, and MC regions. The aging mice had a significantly higher number of lamin A in the hippocampal CA1, CA3, and MC regions. NXP032-treated mice had a significantly reduced number of lamin A in the hippocampal CA1, CA3, and MC regions than in the vehicle-treated mice. In the vit C and Aptamin 320-treated groups, the number of lamin A did not decrease in hippocampal CA1, CA3, and MC regions. The data are presented as the mean ± S.E.M. * *p* < 0.05, compared to the young group. # *p* < 0.05, compared to the aging-vehicle group. Scale bar: 50 μm.

Lamin A accumulates during natural aging as evidence of age-related DNA damage and is activated when cells are exposed to oxidative stress. We evaluated the effect of NXP032 on aging-induced lamin A-positive cells in the hippocampal CA1, CA3, and MC regions using immunohistochemistry (Figure 4). Expression of lamin A-positive cells in the brain was increased in aged mice compared to young mice. However, NXP032-treated mice had significantly decreased expression of lamin A-positive cells in the hippocampal CA1 (F = 2.969, *p <* 0.05), CA3 (F = 11.083, *p <* 0.001), and MC (F = 4.532, *p <* 0.01) regions compared to the aging (vehicle-treated) mice.

### 3.5. NXP032 Suppresses Aging-Induced 4HNE Expression in the Brain

**Figure 5:**
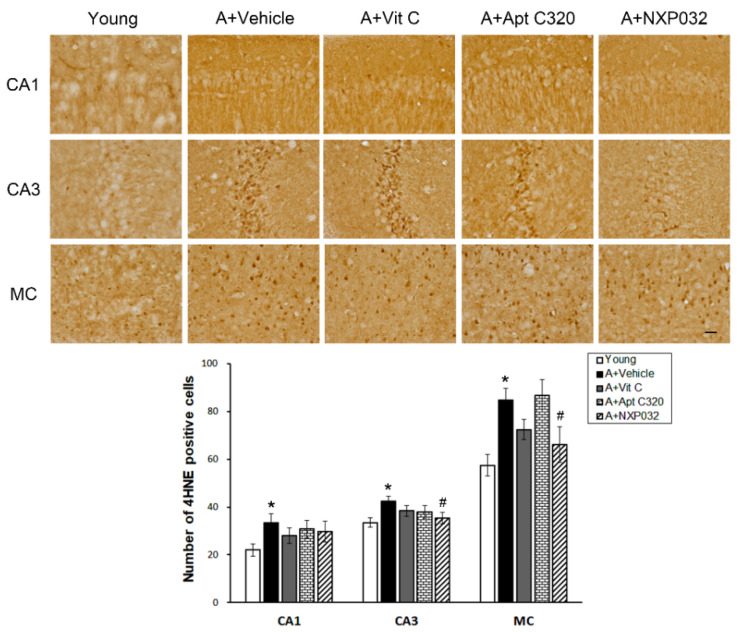
Effect of NXP032 on aging-induced 4HNE production in the hippocampal CA1, CA3, and MC regions. Representative photographs of 4HNE immunostaining in the hippocampal CA1, CA3, and MC regions. The aging mice had a significantly higher number of 4HNE in the hippocampal CA1, CA3, and MC regions. NXP032-treated mice had a significantly reduced number of 4HNE in the hippocampal CA3, and MC regions than in the vehicle-treated mice. In the vit C and Aptamin 320-treated groups, the number of 4HNE did not decrease in hippocampal CA1, CA3, and MC regions. The data are presented as the mean ± S.E.M. * *p* < 0.05, compared to the young group. # *p* < 0.05, compared to the aging-vehicle group. Scale bar: 50 μm.

4-HNE is an oxidative stress marker produced by lipid peroxidation, which accumulates in aging cells and is related to age-related diseases such as neurodegenerative diseases. We evaluated the effect of NXP032 on aging-induced 4HNE positive cells in the hippocampal CA1, CA3, and MC regions using immunohistochemistry (Figure 5). Aging mice had significantly more 4HNE positive cells in the brain compared to young mice. NXP032-treated mice did not have more 4HNE positive cells in the hippocampal CA1 region compared to vehicle-treated mice (F = 1.779, *p =* 0.135). However, in NXP032-treated mice, the number of 4HNE-positive cells was significantly reduced in the hippocampal CA3 (F = 2.119, *p <* 0.05) and MC (F = 5.194, *p <* 0.001) regions compared to in vehicle-treated mice.

### 3.6. NXP032 Increases Aging-Induced γH2AX Expression in the Brain

**Figure 6:**
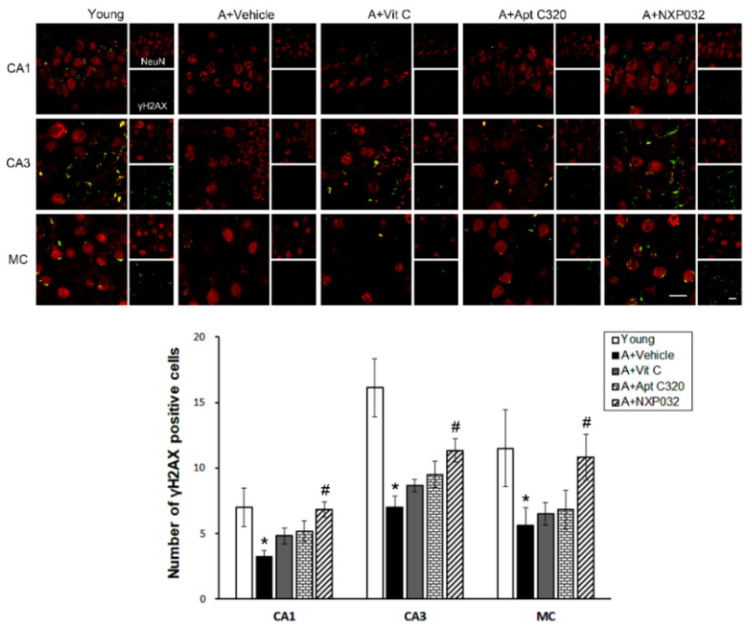
Effect of NXP032 on aging-induced γH2AX expression in the brain. Representative photographs of immunofluorescence staining of γH2AX (green) and NeuN (red) in the brain. The aging mice had a significantly reduced number of γH2AX in the hippocampal CA1, CA3, and MC regions. NXP032-treated mice had a significantly higher number of γH2AX in the hippocampal CA1, CA3, and MC regions than in the vehicle-treated mice. In the vit C and Aptamin 320-treated groups, the number of γH2AX did not increase in hippocampal CA1, CA3, and MC regions. The data are presented as the mean ± S.E.M. * *p* < 0.05, compared to the young group. # *p* < 0.05, compared to the aging-vehicle group. Scale bar: 20 μm.

The level of phospho-histone 2AX (γH2AX) is a marker of DNA damage and is closely related to DNA damage repair. Aging mice had significantly decreased γH2AX expression in the hippocampal CA1, CA3, and MC regions compared to young mice (Figure 6). However, in NXP032-treated mice, the expression of γH2AX was significantly increased in the hippocampal CA1 (F = 2.725, *p* < 0.05), CA3 (F = 7.360, *p* < 0.001), and MC (F = 3.036, *p* < 0.05) regions compared to vehicle-treated mice.

### 3.7. NXP032 Upregulates Nrf2-Keap1 Expression in the Brain

**Figure 7:**
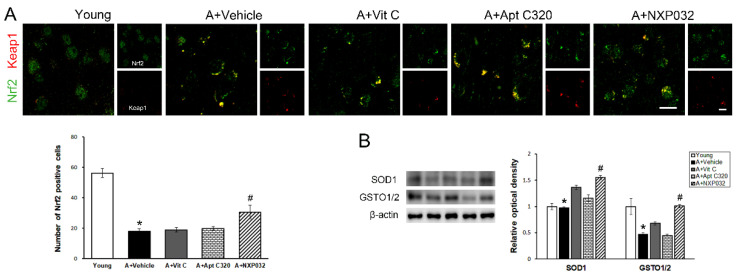
Effect of NXP032 on aging-induced Nrf2 dysfunction in the brain. (**A**) Representative photographs of immunofluorescence staining of Nrf2 (green) and Keap1 (red) in the brain. The aging mice had a significantly reduced number of Nrf2 in the brain. NXP032-treated mice had a significantly higher Nrf2 in the brain than in the vehicle-treated mice. In the vit C and Aptamin 320-treated groups, the number of Nrf2 did not increase in the brain. (**B**) Representative bands of SOD-1 and GSTO1/2 protein expression in the hippocampus. The aging mice had significantly reduced SOD-1 and GSTO1/2 protein expression in the hippocampus. NXP032-treated mice had a significantly higher SOD-1 and GSTO1/2 expression in the hippocampus than in the vehicle-treated mice. In the vit C and Aptamin 320-treated groups, the expression of SOD-1 and GSTO1/2 did not increase in the hippocampus. The data are presented as the mean ± S.E.M. * *p* < 0.05, compared to the young group. # *p* < 0.05, compared to the aging-vehicle group. Scale bar: 20 μm.

Nrf2 is associated with the regulation of cellular defense mechanisms and protection against oxidative stress by regulating the transcription of various antioxidant genes. In the aging group, Nrf2 was mainly located in the cytoplasm, and the expression of Nrf2 was decreased compared to in the young group (F = 36.75, *p <* 0.001). In contrast, NXP032 increased the expression of Nrf2, which was reduced by aging (Figure 7A). To evaluate the expression level of antioxidant enzymes along with the effect of Nrf2 signaling, an important regulator of oxidative stress, we confirmed the expression levels of SOD-1 and GSTO1/2 using Western blotting. Expression levels of SOD-1 (F = 8.85, *p* < 0.001) and GSTO1/2 (F = 6.39, *p* < 0.001) were significantly higher in the NXP032-treated group compared to the vehicle-treated group (Figure 7B). The vit C and Aptamin C320-treated groups did not increase the expression of antioxidant enzymes.

## 4. Discussion

We investigated the effect of a new vitamin C/DNA aptamer complex, NXP032, on aging-induced oxidative stress and cognitive impairment. NXP032 increases antioxidant enzyme activity by enhancing the Nrf2-ARE pathway, reducing aging-induced reactive microglia and astrocytes, DNA damage, and oxidative stress, resulting in neuroprotective effects and cognitive function improvements.

Several studies have shown that cognitive alternations are a part of the normal aging process, including decreased performance in memory tasks that require speed processing or transformation of information to make decisions, such as working memory and executive cognitive functions [25,26]. Aging-induced cognitive impairment and a decrease in brain function may be the result of numerous factors, including changes in neurotransmitters, neuronal damage, and toxic substances that accumulate in the brain over time. Dröge and Schipper’s study demonstrated that aging-induced brain function is associated with an imbalance between antioxidant defenses and intracellular reactive oxygen species through increased oxidative damage due to increased lipid peroxidation, protein oxidation, and DNA oxidation products [27].

The decline in cognitive and motor functions is one of the hallmarks of aging and includes changes in learning and memory associated with the hippocampus, as well as decreased muscle strength, balance, and coordination [25,28]. The mice are a common model for studying aging and aging-related diseases such as dementia and Alzheimer’s disease for a long time. Sauer [29] showed that 18- to 19-month-old mice showed impaired acquisition and retention in the Morris water maze test and decreased cholinergic neurons in the medial septal nucleus. In the present study, 17-month-old mice (aged mice) had cognitive impairment compared to 6-week-old mice (young mice) in the radial 8-arm maze and novel location recognition tests (Figure 1). We confirmed that there were no differences in motor function in behavioral experiments with aged and young mice using an open field test (Supplementary Figure S1). The vit C and Aptamin C320 treatment groups did not have improved cognitive impairment compared to the vehicle-treated aging group. We showed that NXP032 treatment ameliorated aging-induced cognitive impairment through several behavioral experiments. In aged mice, decrease of neuronal cell numbers in the hippocampus and motor cortex was associated with cognitive decline, and NXP032 alleviated the decrease of neurons induced by aging. We have previously conducted similar studies. In our previous study, NXP031 treatment ameliorated cognitive impairment induced by activation of the antioxidant response element (ARE) pathway in a chronic cerebral hypoperfusion model [30].

Age-related neuronal damage activates the expression of reactive genes in microglia and astrocytes [31]. Microglia are immune cells in the central nervous system and play an important role in the removal of cellular debris and useless components, and coordinating and maintaining cerebral homeostasis. Microglial activation can be caused by neuroinflammation and neuronal damage in the central nervous system. Astrocytes play a variety of roles, including structural support, removal of cellular debris, and major constituents of the blood–brain barrier. Reactive astrocytes occur in response to stressful situations in the brain, such as in several neurodegenerative diseases. Aged mice increased the hypertrophic morphology of microglia and astrocytes in the brain compared to young mice, and NXP032 treatment significantly suppressed the activation of Iba1+ microglia and GFAP+ astrocytes in the hippocampus.

Lamin A, a major component of the nuclear lamina, is a product observed along with the major splicing factor of the LMNA gene. The involvement of lamin A in the aging process became clear after the discovery of causal mutations in the *LMNA* gene in Hutchinson–Gilford Progeria Syndrome (HGPS). Yoon et al. provided evidence for the involvement of lamin A/C in p16/INK4A expression and cell senescence [32]. They observed that a p53-mediated lamin A/C increase promoted HGPS-like nuclear deformation associated with cellular aging [33]. The abnormal form of lamin A accelerates nuclear blebbing, downregulation of nuclear envelope proteins, and DNA damage accumulation [34]. Abnormal form of lamin A is characterized by nuclear dysfunction such as decreased DNA repair ability, increased telomere shortening, and increased activation of p53, indicating a decrease in cell lifespan due to premature aging [35]. The physiological concentrations of 4HNE are usually very low, but are markedly increased under sustained oxidative stress. An increase in 4HNE was also confirmed in the plasma of patients with AD and was reported to be related to cognitive impairment [36]. Many previous studies show that 4HNE levels are increased in various organs in a model of aging and that many cellular signaling pathways are involved [37]. In the present study, NXP032 inhibited the aging-induced increase of lamin A and 4HNE expression in the hippocampus and motor cortex regions. These results suggest that NXP032 can reduce aging-induced oxidative stress and aging process in the brain.

DNA damage caused by oxidative stress phosphorylates H2AX to promote signal transduction at the site of damage and promote the fixation of repair proteins. Compared to young mice, the expression of γH2AX is decreased in aged mice, and H2AX proteins are downregulated as well [38]. The results of our study showed that γH2AX was decreased in the aged group compared to the young group but was increased in the NXP032 treated group compared to the vehicle-treated group. Another group demonstrated that H2AX phosphorylation is significantly reduced following acute stress in Nrf2-deficient cells, leading to a decrease in total H2AX protein levels [16].

The activity of Nrf2 was investigated to evaluate the antioxidant effects of NXP032. Nrf2 upregulates antioxidant genes to attenuate oxidative stress due to its antioxidant effect; it also exerts a protective effect against oxidative stress-induced pathophysiology [22]. However, it is well known that the adaptive response to oxidative stress decreases with age [39]. The loss of Nrf2 did not alleviate oxidative stress and may lead to an aged phenotype [24]. In our results, Nrf2 expression was decreased in the aging group but was rescued in the NXP032 group. Activation of the Nrf2-ARE pathway increases the level of expression of the endogenous antioxidants SOD and HO-1 in the hippocampus. In our study, the NXP032-treated group was shown to upregulate the antioxidant SOD-1 and GSTO1/2 expression levels by upregulating Nrf2 expression, which was reduced with aging.

Vitamin C (ascorbic acid) is not only nutritionally and commercially available, but is also a stable substance used in beverages and therapeutics. It is well known that vitamin C protects neurons from oxidative damage caused by ROS [19]. However, these antioxidant properties are prone to degradation through oxidation. To address this problem, we developed NXP032, a DNA aptamer that specifically binds to vitamin C and inhibits oxidation. It has been reported that the presence of an aptamer reduces the oxidation rate of vitamin C by more than 50% and sustains the half-life of vitamin C for more than two weeks [40]. We found that long-term treatment with NXP032 for eight weeks could alleviate aging-induced oxidative stress and strengthened the antioxidant defense system by activating the Nrf2-ARE signaling pathway, thereby demonstrating neuroprotective efficacy in the aging brain [41]. In conclusion, this study suggests that NXP032 can be used as a promising therapeutic agent for age-related cognitive impairment through the inhibition of aging-induced oxidative stress induced by activation of the ARE pathway.

## Data Availability

Not applicable.

## Supplementary Materials

The following supporting information can be downloaded at: https://www.mdpi.com/article/10.3390/antiox11010130/s1, Figures S1 and S2. Figure S1 shows the weights of the groups. Figure S2 shows the motor function in the open field test.
