# Supplementary figures and images for: NXP032 Ameliorates Aging-Induced Oxidative Stress and Cognitive Impairment in Mice through Activation of Nrf2 Signaling
Source: Antioxidants (Basel). 2022 Jan 7;11(1):130. doi: 10.3390/antiox11010130 (PMC8772799; doi:10.3390/antiox11010130)

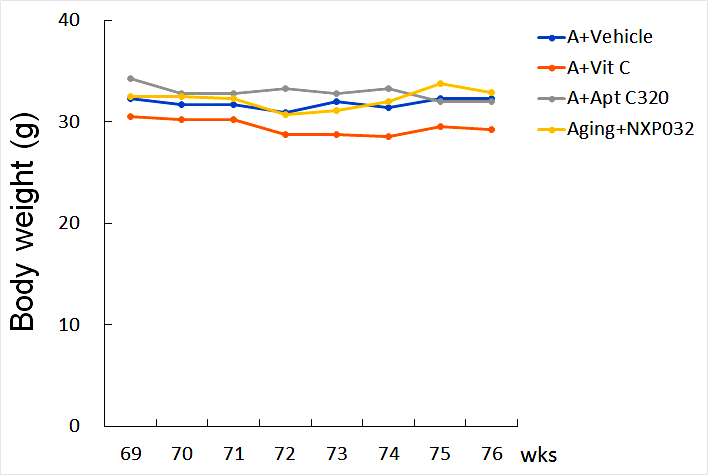

Supplement: Supplementary file 1 [file antioxidants-11-00130-s001.zip › Suppl 1. BW.tif]

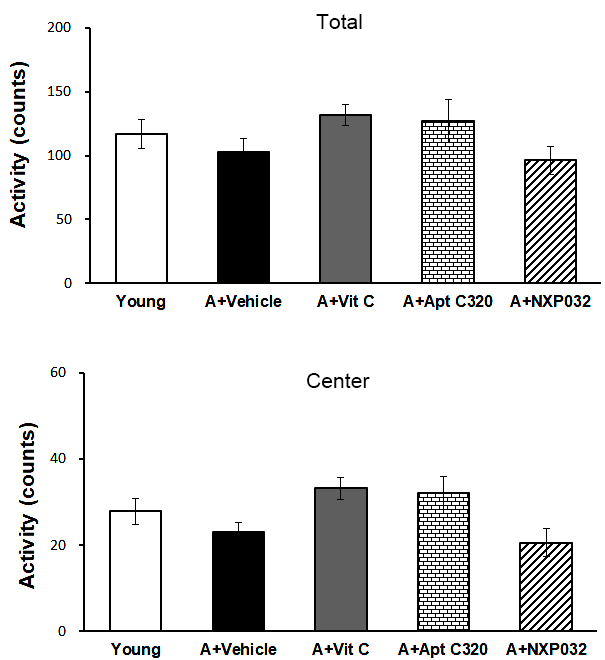

Supplement: Supplementary file 1 [file antioxidants-11-00130-s001.zip › Suppl 2. OFT.tif]
